# Symmetry of Gait in Underweight, Normal and Overweight Children and Adolescents

**DOI:** 10.3390/s19092054

**Published:** 2019-05-02

**Authors:** Veronica Cimolin, Nicola Cau, Alessandro Sartorio, Paolo Capodaglio, Manuela Galli, Gabriella Tringali, Bruno Leban, Micaela Porta, Massimiliano Pau

**Affiliations:** 1 Department of Electronics, Information and Bioengineering, Politecnico di Milano, Piazza Leonardo da Vinci 32, 20133 Milano, Italy; manuela.galli@polimi.it; 2 Orthopaedic Rehabilitation Unit and Clinical Lab for Gait Analysis and Posture, Ospedale San Giuseppe, Istituto Auxologico Italiano, IRCCS, Str. L Cadorna 90, 28824 Piancavallo (VB), Italy; nicola.cau@polimi.it (N.C.); p.capodaglio@auxologico.it (P.C.); 3 Istituto Auxologico Italiano, IRCCS, Experimental Laboratory for Auxo-endocrinological Research, Str. L Cadorna 90, 28824 Piancavallo (VB), Italy; sartorio@auxologico.it (A.S.); gabriella.tringali@yahoo.it (G.T.); 4 Department of Mechanical, Chemical and Materials Engineering, University of Cagliari, Piazza d’Armi, 09123 Cagliari, Italy; bruno.leban@dimcm.unica.it (B.L.); porta.micaela.ib@gmail.com (M.P.); massimiliano.pau@dimcm.unica.it (M.P.)

**Keywords:** gait, accelerometer, spatial-temporal parameters, low disability, harmonic ratio, obesity

## Abstract

Abnormal excess or lack of body mass can influence gait patterns, but in some cases such differences are subtle and not easy to detect, even with quantitative techniques for movement analysis. In these situations, the study of trunk accelerations may represent an effective way to detecting gait anomalies in terms of symmetry through the calculation of Harmonic Ratio (HR), a parameter obtained by processing trunk accelerations in the frequency domain. In the present study we used this technique to assess the existence of differences in HR during gait in a cohort of 75 healthy children and early adolescents (aged 7–14 years) stratified into 3 equally-sized age and gender-matched groups (Underweight: UW; Normal Weight: NW; Overweight: OW). The accelerometric signal, acquired using a single wearable inertial sensor, was processed to calculate stride length, speed, cadence and HR in antero-posterior, vertical and medio-lateral directions. No differences in spatio-temporal parameters were found among groups, while the HR in the medio-lateral direction was found significantly lower in UW children, while OW exhibited the highest values. On the basis of the results obtained, HR appears capable of discriminating gait symmetry in children with different body mass even when conventional gait parameters are unchanged.

## 1. Introduction

Locomotion is a major component of daily physical activities and a key factor in ensuring full functional independence. It is based on a delicate yet sophisticated interplay between the central nervous system and the musculoskeletal system. Among other factors, such balance can be disrupted by weight alterations with respect to physiological status. In particular, either an excess or lack of body mass have been found responsible for specific musculoskeletal adaptations. In particular, excessive mass modifies body geometry by adding passive mass to different regions [1] and it influences the biomechanics of most activities of daily living (ADL), causing functional limitations, increased risk of fall and possibly predisposing to injuries (strain/sprain, lower extremity fracture, and dislocations) [2]. Several studies have addressed the effect of obesity on balance [3], transition from sitting to standing position [4,5] and gait [6,7,8,9], thus unveiling motor abnormalities related to an excess of body mass under static and dynamic conditions. Even an abnormal lack of body mass (i.e., thinness) is likely to originate alterations of basic motor tasks such as gait and postural control [10,11].

It is noteworthy that biomechanical modifications associated with alterations in body mass distribution have been reported mainly in the case of adults, but evidence exists also for children, with particular emphasis on overweight and obesity, which have become a major public health problem [12]. In particular, the walking of overweight children has generally been described as less stable than that of their lean counterparts [13,14,15,16], but a clear pattern of gait alterations of obese children is not evident from the literature analysis. In fact, while several studies have detected significant alterations in spatio-temporal and kinematic parameters of gait in obese children compared to those of normal weight [13,14,15,16], other authors have found differences only in a very restricted set of variables [15,17,18]. A recent review which summarized the results of 25 studies on gait of obese children [19], concluded that there are moderate evidences for increased step width and duration of stance phase, while for all the other spatio-temporal parameters the differences are either non-significant or inconsistent. Even in terms of kinetics, obese children appear to be characterized by gait patterns quite similar to those of normal-weight children, but they are still required to produce greater absolute joint power [20]. This justifies the need for further data to provide a detailed characterization of gait features in individuals with abnormal body mass which, to date, appears far from exhaustive.

In literature, the analysis of parameters derived from trunk accelerations has been demonstrated to be useful to detect subtle alterations of gait patterns. In fact, a number of recent studies have observed that so-called “smoothness of gait” (or, more correctly, “step-to-step symmetry”) can be effectively represented using a parameter (the Harmonic Ratio, HR) which is computed after processing trunk accelerations in the frequency domain. In brief, the HR, calculated for anteroposterior (AP), vertical (V) and mediolateral (ML) directions, quantifies the step-to-step (AP and V direction) or stride-to-stride (ML direction) symmetry being higher values of HRs representative of greater symmetry, and thus providing and an indication of whole body balance during gait [21,22]. In this way, the analysis is not focused on the lower extremity only (as it occurs in most gait analysis techniques such as motion capture systems or electronic walkways), but rather extended to the whole-body through the study of center of mass (COM) accelerations.

Thanks to HRs, additional features related to gait pattern can be seen even when conventional spatio-temporal parameters of gait are not able to reveal specific alterations. Moreover, trunk accelerations are easy to collect with a simple setup (i.e., a single wearable inertial sensor) either in clinical or other ecological settings for walking and thus the measure is not restricted to movement analysis laboratory environments [23,24], which in some cases can be considered a limitation because they require expensive equipment, lengthy set-up, and time-consuming post-processing procedures [25].

Several studies performed in the last two decades have confirmed that such an approach is effective in detecting anomalies of gait in terms of symmetry [21,22,26] and suitable in discriminating gait variations associated with aging [21,23], neurologic disorders [27,28,29] and orthopedic conditions mainly in young and older adults, while scanty applications of this method have so far been reported in children. To the best of our knowledge, only in the study by Chen [30] was HR adopted to assess children with cerebral palsy (CP), with the aim of providing an objective tool for motion disability assessment in clinical diagnosis and rehabilitation therapy of these patients; no studies have been performed in children with body mass alterations.

On the basis of the aforementioned considerations, this study aimed to assess the feasibility of HR as a parameter useful in discriminating differences in gait associated with a lack or excess of body mass in a cohort of healthy children and early adolescents. This topic could be of interest for clinicians and researchers as HR may represent a useful tool to detect the existence of peculiar strategies adopted to achieve a safe and efficient gait even when typical spatio-temporal parameters appear normal. In this way, it would be possible to have available details that may contribute to better assess the effectiveness of physical training, exercises and nutrition programs, particularly important in children and adolescents for lifelong health and well-being and preventing various health conditions.

## 2. Methods

### 2.1. Participants

The study was performed in the period from January 2018 to January 2019 as the result of collaboration between the University of Cagliari (Italy), the Politecnico di Milano (Italy), three primary and secondary schools located in the cities of Cagliari and Elmas (Sardinia, Italy) and the San Giuseppe Hospital, Istituto Auxologico Italiano, IRCCS (Piancavallo, Verbania, Italy). The latter organization, which served as the main recruitment center for obese participants (OW) provided data for 25 individuals (13 males and 12 females). From an overall sample of 280 children and early adolescents aged 7–14 years, screened in the aforementioned schools, two equally sized groups of Normal Weight (NW) and underweight (UW) participants matched for sex, age and height (to limit the effect of possible confounding factors known to influence gait [17,31] were composed. The assignment of the children to each group was carried out on the basis of their Body Mass Index (BMI = weight/height^2^), according to the cut-off points defined by Cole et al. [32,33]. Thus, the analyzed cohort consisted of 75 participants, whose anthropometric features are shown in Table 1.

At the time of testing, none of the children or parents reported any pathological foot abnormalities or other musculoskeletal/neurologic and/or cardiopulmonary condition likely to influence level walking. The underweight and normal weight participants showed normal flexibility and muscle strength; obese subjects were not involved in regular physical activities before hospitalization (less than 1–2 h/week). None of them suffered from diabetes and hypertension, pain, headaches, balance disorders and/or any other symptoms hampering the execution of the tests.

The experimental procedure was explained in detail to children and the study was carried out in accordance with the ethical standards of the institutes and with the 1964 Helsinki declaration and its later amendments and was approved by the ethics committee of the AOU Cagliari (authorization no. PG/2015/16965). All participants and their parents (or legal guardians) signed an informed consent form. In order to ensure adequate level of confidentiality, the results of the study were disclosed to parents only after asking for permission of the child [34].

### 2.2. Data Acquisition

Gait analysis was performed using a single wearable inertial sensor (G-Sensor^®^, BTS Bioengineering, Italy), previously employed in studies of gait in children [35,36]. It was attached to the participant’s trunk (at L4-L5 vertebrae level) using a semi-elastic belt. The device measures acceleration along three orthogonal axes (antero-posterior AP, medio-lateral ML, and supero-inferior V) and transmits them via Bluetooth to a PC. Following a verbal start signal, participants walked along a 30-m hallway at a self-selected comfortable speed. Acceleration data were acquired at a frequency of 100 Hz for at least twenty consecutive strides performed in the central part of the walking pathway. Such conditions (i.e., walking distance and minimum number of acquired strides) are consistent with the suggestions provided by most previous similar studies as summarized by Pasciuto et al. [37]. Overall, two trials were performed: the first served for familiarization purposes while the second was actually acquired.

The accelerometric signal was processed with a custom Matlab^®^ routine to calculate:

(1) spatio-temporal parameters of gait (namely gait speed, stride length and cadence);
(2) HRs, which refer to AP (i.e., direction of motion), ML and V directions.

In particular, the spatio-temporal parameters are defined as follows:

(1) Gait speed: the mean velocity of progression (m/s);
(2) Stride length: the longitudinal distance between successive ground contacts of the same foot (m);
(3) Cadence: the rate at which a person walks (steps per minute).

The spatio-temporal parameters were calculated in the basis of the approach described by Cimolin et al. [9], which was already successfully employed also on obese individuals. Instead, as regards the HRs computation, two procedures of calculation were implemented: the first one, which is the most widespread, was described and extensively tested by Menz et al. [26]. In brief, the accelerometric signal is firstly processed in the frequency domain using a finite Fourier series, then the HR (for AP and V direction) is calculated as ratio between the sum of the amplitudes of the first ten even harmonics by the sum of the amplitudes of the first ten odd harmonics. The even harmonics indicate the in-phase components of the signal, while the odd harmonics refer to the out-of-phase components, the latter being minimized as gait symmetry improves. As regards the ML direction, since the acceleration pattern exhibit one peak per stride, (resulting in dominance of the first harmonic and subsequent odd harmonics) the calculation of HR is carried out by dividing the sum of the amplitudes of the odd harmonics divided by the sum of the amplitudes of the even harmonics.

Recently, Pasciuto et al. [37] revised this approach by introducing the so called “improved HR” (iHR), which was thought to overcome some limitations of the HR, namely a large variability even for highly symmetrical gait and a certain difficulty in its interpretation, as traditional HR may theoretically range from 0 (complete asymmetry) to infinite (perfect symmetry). The iHR is calculated by dividing the square of the even harmonics (AP and V directions) or odd harmonics (ML direction) by the sum of the squares of even and odd harmonics and multiplying this ratio for 100. In this way, the ratio is normalized obtaining an index which ranges from 0 (total asymmetry) to 100 (total symmetry) and thus is easier to interpret.

The differences introduced in spatio-temporal and HRs parameters by lack/excess of body mass were assessed using a one-way multivariate analysis of variance (MANOVA) setting as the independent variable the participant’s status (UW, NW or OW), while the dependent variables were respectively the 3 spatio-temporal parameters and the 3 HRs. The level of significance was set at *p* = 0.05. Univariate ANOVA was carried out as a post-hoc test by reducing the level of significance to *p* = 0.016 (0.05/3 for both spatio-temporal parameters and HR) after a Bonferroni correction for multiple comparisons.

The degree of correlation between BMI and HR was then investigated using Spearman’s rank correlation coefficient rho by setting the level of significance at *p* = 0.05 even in this case. The rho values of 0.1, 0.3, and 0.5 were considered representative of small, moderate, and large correlations respectively, according to Cohen’s guidelines [38]. All analyses were carried out using the IBM SPSS Statistics v.23 software (IBM, Armonk, NY, USA).

## 3. Results

**Figure 1:**
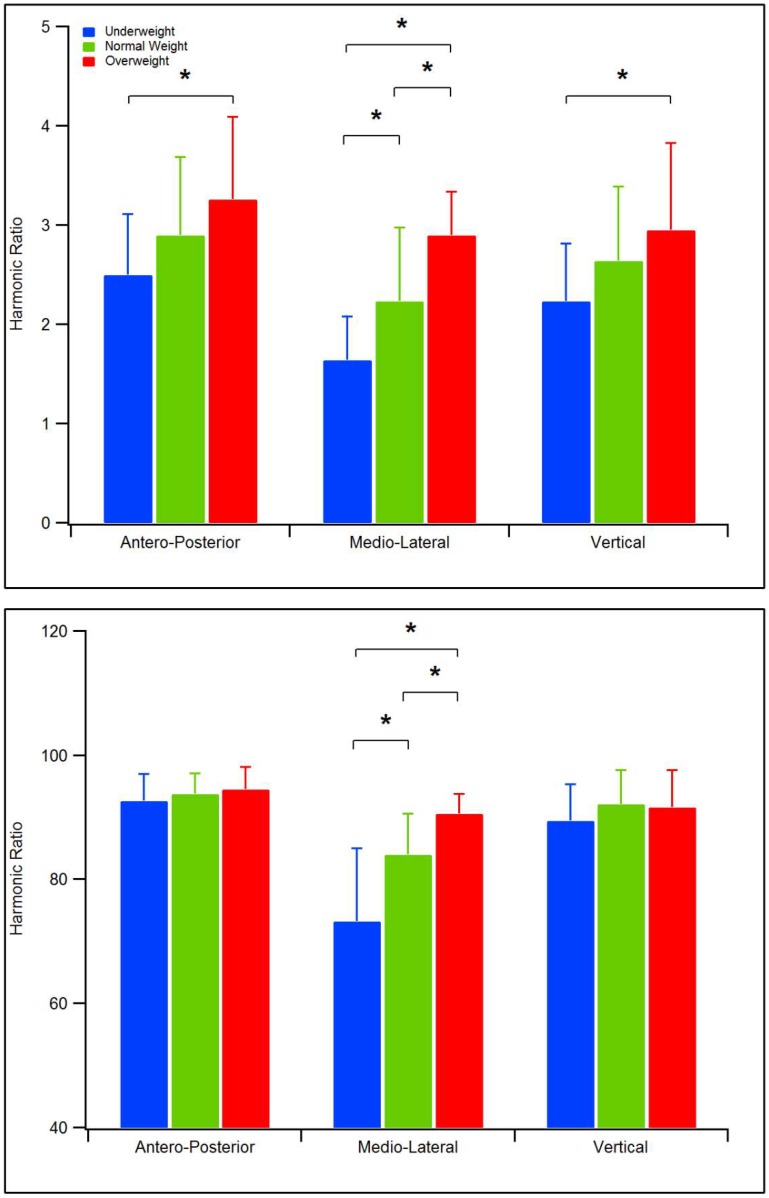
Trend of HR values for underweight, normal weight and overweight children. Top: values calculated according to the procedure described by Menz et al. [26]. Bottom: values calculated according to the procedure described by Pasciuto et al. [37].

The results of the experimental tests are summarized in Table 2 and Figure 1.

No main significant group effect was detected by MANOVA as regards the spatio-temporal parameters [F(6,140) = 1.50, *p* = 0.181, Wilks *λ*= 0.88, *η*^2^ =0.06], while body mass was found to significantly impact symmetry of gait measures [F(6,140) = 9.07, *p* < 0.001, Wilks *λ*= 0.52, *η*^2^ =0.28] for the calculation of HR according to Menz et al. [26] and [F(6,140) = 8.73, *p* < 0.001, Wilks *λ*= 0.53, *η*^2^ = 0.27] for improved HR proposed by Pasciuto et al. [37]. In particular, the follow-up ANOVA showed the existence of significant differences between all three groups as regards the HR in the ML direction, with underweight children who exhibit the lowest values (1.64 vs. 2.23 of NW and 2.90 of OW, using the Menz approach, and 73.2 vs. 83.98 of NW and 90.58 of OW, with the Pasciuto method, *p* < 0.001 in all cases). Underweight children also exhibited the lowest HR values in the AP and V directions, with a significant difference in comparison with OW participants.

The results of the correlation analysis (reported in Table 3) revealed the existence of large (for HR in the ML direction) and moderate (HR in the AP and V directions) significant positive correlations between BMI and symmetry of gait parameters when calculated using the Menz et al. [26] approach. The magnitude of the correlation was confirmed for the ML direction even when the improved HR [37] was considered. However, for the V direction the relationship between BMI and HR was no longer significant and for the AP direction the rho value was found slightly lower with respect to the Menz calculation.

## 4. Discussion

In this study, we investigated the existence of possible variations in HR values associated with different gait strategies of children and adolescents characterized by significant body mass alterations. The HR of the trunk acceleration series seems to represent a valid measure of walking symmetry under different conditions and recently there has been increasing interest in applying this technique to discriminate between individuals with different pathologies and to monitor the impact of rehabilitation protocols [22].

The findings of the present study indicate that despite the similarity in terms of main spatio-temporal parameters of the three groups investigated (which are known to have a direct influence on symmetry parameters [26]), some interesting differences were observed in their HR values, particularly as regards the ML direction. In fact, our data suggest that underweight children exhibit a significant reduction in walking symmetry with respect to both normal weight and obese children, the latter showing the highest HR values. Although there are few studies that employed HR to investigate gait alterations in presence of excessively high or low body mass, it is noteworthy that our findings are consistent with those of Misu et al. [39], who reported that in presence of malnutrition, older adults exhibited lower values of HR in the ML direction with respect to normal weight individuals. This suggests that walking symmetry in the ML direction sensitively reflects some of the deficits in gait performance caused by thinness. Maintaining equilibrium during walking requires continuous integrative control, particularly in the ML direction due to inherent instability during single limb support [40]; thus, according to literature [39] and our findings, thinness, which characterized our UW group, might decrease gait steadiness in children.

In contrast, it was quite surprising to observe that overweight children exhibit significantly higher values of HR in the ML direction, even with respect to normal weight peers. According to the accepted interpretation of HR, this would indicate a superior symmetry, which is supposed to indicate a better rhythmic pattern of acceleration and deceleration of the trunk during walking [23] and a higher dynamic balance. In this case, there are no available data to compare with, but some indirect evidence may explain this phenomenon. There is a certain consensus in the literature on the fact that obese individuals walk with a significantly increased base of support (i.e., larger step width with respect to normal weight) [15,41], in order to reach and guarantee a higher dynamic stability. Larger step width has been found associated with increased values of medial lateral components of the GRF [42], which are also typical features of gait in obese individuals [41,43]. Since acceleration in the ML direction is influenced by the ML component of the GRF (that is, increased GRF originates major accelerations) we can hypothesize that the observed increase in HR in ML direction is directly linked to such phenomenon. The higher HR value, and thus higher gait symmetry, in ML direction may be connected to the search for better gait stability, increasing the rhythmic pattern of acceleration and deceleration of the trunk during walking [23]. In addition, the increase in step width contributes to ML dynamic equilibrium, and this is likely to be positively reflected in those aspects of gait (rhythm, symmetry and stride-to-stride variability) which are encompassed in the HR ML [44]. The important role played by HR ML as a determinant of stability is also confirmed by previous studies reporting that risk of falls is associated with this parameter [44,45].

**Figure 2:**
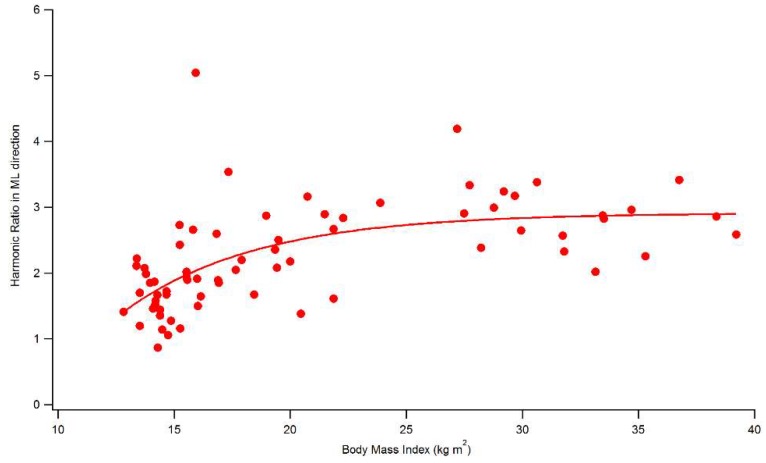
Trend of HR in the ML direction (calculated according to Menz et al., 2003 [26]) with the BMI for all participants.

In Figure 2 is shown the trend of HR in ML direction with increasing BMI, as computed according to Menz et al., [26]. Interestingly, it is possible observe a positive strong relationship (Spearman’s rho = 0.672, *p* < 0.001) between HR and BMI, which is markedly nonlinear. In particular, the curve exhibits a steeper gradient for BMI values approximately in the range 12–20 kg/m^2^ while for higher BMI a sort of plateau is reached. This would lead to hypothesize the presence of a sort of cut-off in terms of BMI that separates a region of HR more sensitive to even slight BMI variations, from one in which even relevant changes in BMI do not appear able to greatly affect HR. Further research performed on a larger sample should be conducted to identify the BMI cut-off value.

The HR values were calculated using two different approaches. The first (proposed by Menz et al. [26]) is the most widespread and thus a large amount of data for different experimental conditions are available for comparison purposes. Recently, Pasciuto et al. [37] introduced a technique to calculate the so-called “improved” HR, which the authors demonstrated to be more robust with respect to the number of harmonics and strides considered, and characterized by a lesser inter-stride variability. Application of the two methods to the acceleration data of our sample provided unanimous results only as regards HR in the ML direction, and thus we can reasonably hypothesize that the observed differences are actually associated with the BMI differences of the three groups tested. The HR data obtained with the Menz algorithm were instead found significantly different in UW and OW participants for both the AP and V directions, while this was not true in the case of the Pasciuto method. While this can be due to a different sensitivity of the two approaches, it is noteworthy that the Pasciuto method is very recent and there are still scarce data to refer to. Moreover, the authors tested it only in two cases (elderly woman and trans-femoral amputees vs. young adults) on a limited number of samples. However, given the differences observed between the two calculation methods for AP and V directions, at the moment we cannot be sure that their HR are actually influenced by body mass only, and thus further investigations are needed to clarify this issue.

However, on the basis of the data obtained, we can state that the HR calculated from trunk acceleration and successfully used in previous studies in young and elderly adults, could represent a useful complement to the conventional spatio-temporal parameters of gait as it is able to characterize subtle alterations in locomotor mechanisms not recognized by the typical spatio-temporal gait. Then, HR could be useful to have a point of view on the gait not focused to lower extremities, but rather on COM, and then have a global view of the body movement. In addition, this study represents the first attempt in applying HR computation in children, and the results seem to be encouraging. Walking symmetry could be used in the future to investigate the existence of changes in the quality of trunk control during walking due to growth and to assess the effects of rehabilitative interventions and weight loss, specifically for overweight and obese children. This approach requires minimal equipment (a single accelerometer attached to the trunk) and is characterized by several benefits: (a) it is relatively low-cost (compared with a gait laboratory), (b) data collection is not restricted to a laboratory setting, (c) data can be collected over an extended walking distance and over various grounds and conditions, and (d) the accelerometer is relatively small and unobtrusive, so it does not interfere with walking.

As this study focused solely on symmetry, we were able only to speculate on the mechanisms underlying the pattern of results. A future direction of research would be to examine kinematic and kinetic data together with HRs and other trunk acceleration-derived measures to better understand how the system controls global body motion during walking, and which measures are most sensitive to weight-related changes in gait. In addition, the further analysis of kinematics and kinetics may explain where the deviation from symmetry occurs [22]. Walking acceleration is in fact a complex combination of phase and amplitude of the harmonic components, and HRs are a global measure that do not account for phase. Thus, different pathologies may affect phase differently, yet result in the same HRs. In addition, HRs are demonstrated to be speed-dependent and in particular speed was found a significant contributor to HRs in the very slow condition [21]. Our data showed no statistical differences among assessed groups in terms of velocity, even if a slight trend of speed increase was found in OW participants respect to the others participants. Nevertheless, we suppose that the speed increase is really modest (+15% respect to UW and NW children) to justify a significant HR increase in ML direction (+79% respect to UW individuals and +27% respect to NW individuals). Further investigation should be conducted in this direction, evaluating with larger samples the effects of speeds faster and slower than preferred on the HRs.

Moreover, although we detected no difference in any of the investigated spatio-temporal parameters of participants, it must be considered that we were unable to assess step width, which is not measurable with an inertial sensor. Step width was found increased in obese individuals [15,41] and thus it may represent an early sign of the presence of gait alterations. Obviously for a more in-depth assessment of gait strategy, the inertial sensor cannot fully replace movement analysis laboratories based on optoelectronic systems. However, the information obtained by a single sensor provides low-cost quantitative gait parameters with minimal interference with a subject’s habitual tasks outside the laboratory.

Some limitations of the study should be acknowledged: at first, since the size of the sample here tested was quite reduced, further studies should be carried out on a larger number of participants. This would allow to better understand important aspects of the phenomenon such as, for example, the existence of different trends of HR for boys and girls [46], which might be associated to the way fat mass is distributed across the body. Also, in our study we tested only level walking and straight trajectories that children traveled at self-selected speed. As previous studies reported that irregularities in trajectory and surface, as well as speed are able to influence HR values [21,23,26], a range of different (and possibly more ecological) conditions should be tested to verify whether the differences in symmetry here observed remains or change. On the other hand, from a clinical point of view, the use of HR to characterize subtle aspects of gait in children characterized by abnormal BMI has several advantages like low cost, possibility to collect data over a wide range of walking distance and surface conditions, and negligible intrusivity, this latter fact essential to avoid interference of the measure process with walking.

## 5. Conclusions

Although relevant excess or lack of body mass in children and early adolescents seems not to affect significantly the main spatio-temporal parameters, the trunk accelerations, properly processed to calculate HR, revealed significant differences in terms of step-to-step and stride-to-stride symmetry. In particular, it was observed a trend of increase for HR in all the considered directions, even though the most significant results are those associated with the ML direction. Such findings suggest that BMI influences, to some extent, the whole-body movement, and thus HR may represent a useful tool to investigate subtle changes in gait dynamics when other conventional parameters fail in detect any differences.

## Figures and Tables

**Table 1:** Demographic and anthropometric characteristics of participants. Values are expressed as mean ± SD.

	Underweight	Normal Weight	Overweight
Participants # (M,F)	25 (13M, 12F)	25 (13M, 12F)	25 (13M, 12F)
Age (years)	11.1 ± 1.0	11.2 ± 1.2	11.7 ± 1.8
Height (cm)	148.2 ± 9.9	147.8 ± 10.3	151.9 ± 14.6
Body Mass (kg)	31.6 ± 5.2	38.9 ± 7.7	71.5 ± 21.4 ^a,b^
Body Mass Index (kg·m^−2^)	14.3 ± 0.7	17.6 ± 1.9 ^a^	30.2 ± 5.4 ^a,b^

^a^ significant difference vs. Underweight (*p* < 0.001); ^b^ significant difference vs. Normal weight (*p* < 0.001).

**Table 2:** Spatio-temporal and symmetry-of-gait parameters calculated for underweight, normal weight and overweight participants. Values are expressed as mean ± SD.

		Underweight	Normal Weight	Overweight
Spatial-temporal parameters of gait	Gait speed (m·s^−1^)	1.23 ± 0.29	1.22 ± 0.30	1.41 ± 0.39
	Stride length (m)	1.18 ± 0.32	1.18 ± 0.23	1.27 ± 0.46
	Cadence (steps min^−1^)	127.59 ± 23.01	123.72 ± 18.35	146.13 ± 65.01
Symmetry of gait (HR, Menz et al. [26])	AP direction	2.50 ± 0.80	2.90 ± 0.79	3.25 ± 0.83 ^a^
	ML direction	1.64 ± 0.44	2.23 ± 0.74 ^a^	2.89 ± 0.44 ^a,b^
	V direction	2.23 ± 0.59	2.64 ± 0.75	2.95 ± 0.88 ^a^
Symmetry of gait (iHR, Pasciuto et al. [37])	AP direction	92.68 ± 4.34	93.74 ± 3.35	94.53 ± 3.64
	ML direction	73.20 ± 11.80	83.98 ± 6.62 ^a^	90.58 ± 3.22 ^a,b^
	V direction	89.44 ± 5.90	92.09 ± 5.44	91.59 ± 6.01

^a^ significant difference vs. Underweight after Bonferroni correction (*p* < 0.016); ^b^ significant difference vs. Normal Weight after Bonferroni correction (*p* < 0.016).

**Table 3:** Spearman’s rho correlation coefficients between BMI and HR values.

		Menz et al. (2013)	Pasciuto et al. (2017)
BMI vs.	HR AP direction	0.370 **	0.252 *
	HR ML direction	0.672 **	0.663 **
	HR V direction	0.375 **	0.213

* *p* < 0.05; ** *p* < 0.01.
